# Biological function of Lemur tyrosine kinase 2 (LMTK2): implications in neurodegeneration

**DOI:** 10.1186/s13041-018-0363-x

**Published:** 2018-04-10

**Authors:** János Bencze, Gábor Miklós Mórotz, Woosung Seo, Viktor Bencs, János Kálmán, Christopher Charles John Miller, Tibor Hortobágyi

**Affiliations:** 1 Division of Neuropathology, Institute of Pathology, Faculty of Medicine, University of Debrecen, Nagyerdei krt. 98., Debrecen, H-4032 Hungary; 2 Department of Basic and Clinical Neuroscience, Institute of Psychiatry Psychology and Neuroscience, King’s College London, London, UK; 3 Department of Psychiatry, Faculty of Medicine, University of Szeged, Szeged, Hungary; 4 MTA-DE Cerebrovascular and Neurodegenerative Research Group, Debrecen, Hungary; 5 Department of Pathology, Faculty of Medicine, University of Szeged, Szeged, Hungary; 6 Department of Old Age Psychiatry, Institute of Psychiatry Psychology and Neuroscience, King’s College London, London, UK

**Keywords:** Alzheimer’s disease, Axonal transport, LMTK2, Neurodegeneration, Tau

## Abstract

Neurodegenerative disorders are frequent, incurable diseases characterised by abnormal protein accumulation and progressive neuronal loss. Despite their growing prevalence, the underlying pathomechanism remains unclear. Lemur tyrosine kinase 2 (LMTK2) is a member of a transmembrane serine/threonine-protein kinase family. Although it was described more than a decade ago, our knowledge on LMTK2’s biological functions is still insufficient. Recent evidence has suggested that LMTK2 is implicated in neurodegeneration. After reviewing the literature, we identified three LMTK2-mediated mechanisms which may contribute to neurodegenerative processes: disrupted axonal transport, tau hyperphosphorylation and enhanced apoptosis. Moreover, *LMTK2* gene expression is decreased in an Alzheimer’s disease mouse model. According to these features, LMTK2 might be a promising therapeutic target in near future. However, further investigations are required to clarify the exact biological functions of this unique protein.

## Introduction

Neurodegeneration is characterised by irreversible structural and functional damage of neurons leading to extensive cell death in numerous central nervous system disorders. According to the affected central nervous system regions, a wide range of clinical symptoms (e.g. dementia, movement disorder, etc.) can be observed. In definition, dementia is an acquired, progressive cognitive decline severe enough to make difficulties in daily-life [1]. Alzheimer’s disease is the most frequent neurodegenerative dementia with a prevalence of 26.6 million [2]. There are several and relatively common neurodegenerative disorders (e.g. amyotrophic lateral sclerosis) where the neuronal damage affects other region of the central nervous system (i.e. motor neurons) although some forms of motor neuron diseases are also accompanied by cognitive impairments and dementia [3, 4]. Considering the fact that clinical symptoms usually appear in elderly, except in uncommon familial forms, prevalence will undoubtedly increase in the next few decades. Prince et al. have predicted that the number of patients with dementia will double every 20 years reaching 115.4 million globally by 2050 [5]. This tendency in our aging society may raise dementia to the most challenging public health issue for the medical and social care system in the future. Despite their significance, neurodegenerative diseases are still incurable. The available therapies are limited to mitigation and mere delay of clinical symptoms. Therefore, intensive study of the field is essential to reveal the underlying pathomechanism and to identify new therapeutic targets for drug development. There is emerging evidence that alterations in the level of synaptic proteins and in their regulation are highly involved in the molecular pathogenesis [6–9], and clinical symptoms [10–12] of neurodegenerative disorders.

LMTK2, a brain enriched neuronal kinase has recently become of interest in neurodegenerative disease research since it regulates a number of fundamental cellular pathways linked to neurodegeneration. These include links to cyclin-dependent kinase 5 (CDK5)/p35, glycogen synthase kinase-3β (GSK3β), protein phosphatase-1 (PP1) and also the axonal transport machinery [13–16]. Therefore, changes in LMTK2 expression and activity may disrupt synaptic regulatory processes and axoplasmic flow to synapses leading to synaptic dysfunction, and neurodegeneration. Although our current knowledge on the biological functions of LMTK2 is limited compared to many other kinases, we aimed to provide a brief review of the literature and to delineate LMTK2’s potential role in neurodegenerative pathology.

## Characteristics of LMTK2

LMTK2 was originally described by three groups independently using yeast two-hybrid screens and database search [14, 16, 17]. The gene of *LMTK2* has been mapped on human chromosome 7q21.3 and encodes a protein with molecular weight of 250 kDa. Due to parallel discoveries, LMTK2 is also known as kinase/phosphatase/inhibitor-2, CDK5/p35-regulated kinase, brain-enriched kinase, apoptosis-associated tyrosine kinase-2 and KIAA1079 [14, 16–19]. LMTK2 is a member of the structurally unique membrane anchored lemur kinase protein family along with LMTK1A, LMTK1B and LMTK3 [18, 19]. At first sight, LMTK2 appears to be a dual specific tyrosine-serine/threonine kinase based on the similarity of its kinase domain sequence to other tyrosine kinases. However, it has been demonstrated by independent studies that LMTK2 is a serine/threonine-specific kinase and do not target tyrosine residues [14, 17, 20]. Therefore, the name ‘lemur tyrosine kinase’ does not accurately reflect the kinase property of this protein. We suggest ‘lemur tail kinase’ as new name instead of the misleading and confusing ‘lemur tyrosine kinase’. In this way, the acronym LMTK can be kept ensuring that the gene/protein naming remains consistent in the literature.

LMTK2's kinase activity was investigated by in vitro kinase assays and peptide microarray. In these experiments, recombinant and highly purified LMTK2 protein was used (i.e. no other kinase was present in the reaction) which exhibited autophosphorylation and also phosphorylated target proteins suggesting that LMTK2 is a constitutively active kinase [14, 20] LMTK2 is anchored into membranes by its tandem amino-terminal transmembrane domains in a way that both its amino- and carboxyl-termini face towards the cytoplasm [21]. The transmembrane domains are followed by an amino-terminal kinase domain and a long carboxyl-terminal ‘tail’. This long ‘tail’ inspired the recently used name after the long-tailed Madagascan lemurs. LMTK2 is predominantly but not exclusively expressed in the brain and highly enriched in the hippocampus and cerebral cortex [16–19]. In neuronal cells, it is located in the cell body, along neurites and in growth cones [16–18]. Intracellularly, a proportion of LMTK2 is present in the Golgi apparatus and early endosomes [16, 22, 23]. LMTK2 starts to be highly expressed in the first two-three postnatal weeks [16–18] suggesting its role in postnatal neuronal development. In support of this notion, LMTK2 is reported to undergo rapid, protein kinase C (PKC)-dependent phosphorylation in PC12 cells following neuronal growth factor (NGF) stimulation however, if PKC directly phosphorylates LMTK2 is not known [17, 20]. NGF signalling reduces LMTK2 activity which in turn enhances neurite outgrowth indicating that LMTK2 is a negative regulator of neuronal differentiation [17, 20]. Another regulator of LMTK2 kinase activity is CDK5/p35 and the details of this regulatory mechanism are discussed in the next section below. Interestingly, the carboxyl-terminal tail of LMTK2 contains seven proline-(any residue-any residue)-proline (PxxP) motifs [17]. Proteins with PxxP motif can directly bind to proteins with Src homology 3 (SH3) domain [24, 25]. Numerous kinases contain SH3 domains or interact with target proteins via SH3 domain containing scaffolding proteins [26, 27]. Therefore, LMTK2 might be regulated by kinases with SH3 domains or alternatively, LMTK2 can phosphorylate downstream targets by recruiting them by its PxxP motifs. Although this is an attractive theory no SH3 domain containing LMTK2 binding partner has been identified yet.

To date, one LMTK2 animal model has been published. LMTK2 knockout mice have been found to be viable but male mice are infertile due to azoospermia [28]. This alteration derives from the defected maturation of germ cells, suggesting that LMTK2 is also essential to physiological spermatogenesis. However, the effects of LMTK2 loss on the nervous system in these animals has not been properly studied and reported. In order to dissect neuronal function of LMTK2, further LMTK2 animal models need to be developed and investigated.

## LMTK2 interacting partners

Although a fully detailed interacting network of LMTK2 remains to be elucidated there are several known protein-binding partners and signalling pathways which suggest essential role of LMTK2 in key cellular processes. LMTK2 interacting partners include CDK5/p35, the catalytic subunit of protein phosphatase 1 (PP1C) and myosin VI. Details of their interaction and potential cellular functions are discussed below.

### CDK5/p35

CDKs are proline-directed serine/threonine protein-kinases involved in cell cycle and transcription regulation, neuronal morphogenesis, and neuronal differentiation [29]. LMTK2 interacts with CDK5 via binding to p35, the activator subunit of CDK5 [15, 16]. This interaction is mediated by a sequence (amino acid 391–632) closely located to the kinase domain of LMTK2 however, where LMTK2 binds to p35 and how this interaction is regulated is not known [16]. CDK5 is a unique member of the CDK family. Unlike other CDKs, CDK5 does not require activating phosphorylation by cyclins instead it needs binding to activator proteins or their cleaved counterparts p35/p25, or p39/p29 [30, 31]. CDK5 has vital role in neuronal maturation, migration, synaptic plasticity and memory formation as well as in synaptic vesicle exocytosis by phosphorylating several downstream targets [29, 31]. CDK5/p35 not only binds to LMTK2 but also phosphorylates it on serine-1418 [15, 16]. This phosphorylation induces increased LMTK2 kinase activity and simultaneously stimulates its ability to phosphorylate downstream targets such as PP1C [15]. Thus, LMTK2 together with CDK5/p35 can regulate key neuronal processes which are vital for proper neuronal functioning. CDK5 is functionally inactive in non-neuronal tissues because CDK5 activator p35 expression is restricted to neurons [15, 32–34]. Nevertheless, a small amount of phospho-LMTK2 serine-1418 was still detectable in non-neuronal cells suggesting that other kinases can also activate LMTK2 by phosphorylating LMTK2 serine-1418 in non-neuronal tissues [15]. Interestingly, both CDK5 and mitogen-activated protein kinase (MAPK) are proline-directed serine/threonine protein-kinases with a very similar consensus sequences which are (any residue)-serine/threonine-proline-(any residue)-lysin/histidine/arginine for CDK5, and proline-(any residue)-serine/threonine-proline for MAPK [35, 36]. This similarity that both CDK5 and MAPK require a proline immediately downstream of the targeted serine/threonine residue raises the possibility that MAPK might also target the same residues in LMTK2 as CDK5. Thus, LMTK2 kinase activity might not only been regulated by CDK5/p35 but also by MAPK. Indeed, experimental data show that treating PC12 cells with NGF or PKC activator increases MAPK/CDK specific phosphorylation of LMTK2 however, if this increased phosphorylation was induced directly by MAPK or CDK5 is not known [17]. To sum up, LMTK2 can phosphorylate and regulate downstream targets, and play important role in both neuronal and non-neuronal processes.

### PP1C

PP1 is a protein serine/threonine phosphatase which is involved in myriad fundamental cellular functions. Interacting with distinct regulatory subunits, PP1C controls cell cycle, apoptosis, glycogen homeostasis, RNA splicing, protein synthesis, muscle activity and neuronal processes [37, 38]. PP1C binds to proteins with an arginine-valine-(any residue)-phenylalanine motif, also known as RVxF motif [39, 40]. LMTK2 contains a valine-threonine-phenylalanine (VTF) motif close to its carboxyl-terminus and binds to PP1C through this motif [13–15]. Upon binding to PP1C, LMTK2 phosphorylates it on threonine-320 which attenuates the phosphatase activity of PP1C [13–15]. This PP1C inhibitory activity of LMTK2 is facilitated by CDK5/p35-dependent phosphorylation of LTMK2 on serine-1418, thus CDK5/p35 and PP1C together with LMTK2 function in one common signalling pathway [13, 15]. Interestingly, LMTK2 also binds to inhibitor-2, one of the regulatory subunits of PP1C which restricts PP1C activity when they interact [14, 41]. Thus, LMTK2 regulates PP1C activity and in this way downstream cellular processes by two independent mechanisms phosphorylating it on threonine-320, and complex it with inhibitor-2.

### Myosin VI

Myosin VI is an actin-based molecular motor protein involved in retrograde transport of endo- and exocytotic membranes. It is known that some binding partners of myosin VI are involved in intracellular targeting and recruitment of myosin VI [42–45]. Two research groups simultaneously identified LMTK2 as a myosin VI binding partner. LMTK2 directly binds to a tryptophan-tryptophan-tyrosine (WWY) motif in the carboxyl-terminal tail of myosin VI [22, 46]. The WWY motif is the same site where other endocytic adaptor proteins bind to myosin VI however, how cargoes are selected and their binding to myosin VI is regulated is not known [42, 46, 47]. LMTK2 binds to the WWY motif of myosin VI through a region close to its kinase domain (amino acid 567–773) which overlaps with the p35 binding region (amino acid 391–632) [16, 22]. This overlap between the residues where myosin VI and p35 associate to LMTK2 raise the possibility of competition between myosin VI and p35 for LMTK2 binding although, to date, there is no confirming experimental data. LMTK2 and myosin VI co-localise in endocytic and recycling endocytic vesicle compartments. Through binding to myosin VI, LMTK2 is essential for the endocytic transport of transferrin receptor, a cargo of myosin VI [22, 23]. Interestingly, cystic fibrosis transmembrane conductance regulator (CFTR), a chloride ion selective ion channel which mutation causes cystic fibrosis, binds not only to myosin VI but also directly interacts with LMTK2 [20, 48–50]. In addition, CFTR is also a substrate of LMTK2. Phosphorylation of CFTR on serine-737 by LMTK2 enhances CFTR endocytosis and thus LMTK2 regulates CFTR availability on the cell surface [20, 48]. These findings indicate that LMTK2 has significant role in the orchestration of endocytic/recycling machinery together with myosin VI. Yet, the exact regulation of this mechanism remains to be elucidated.

## Implications in neurodegeneration

**Fig. 1:**
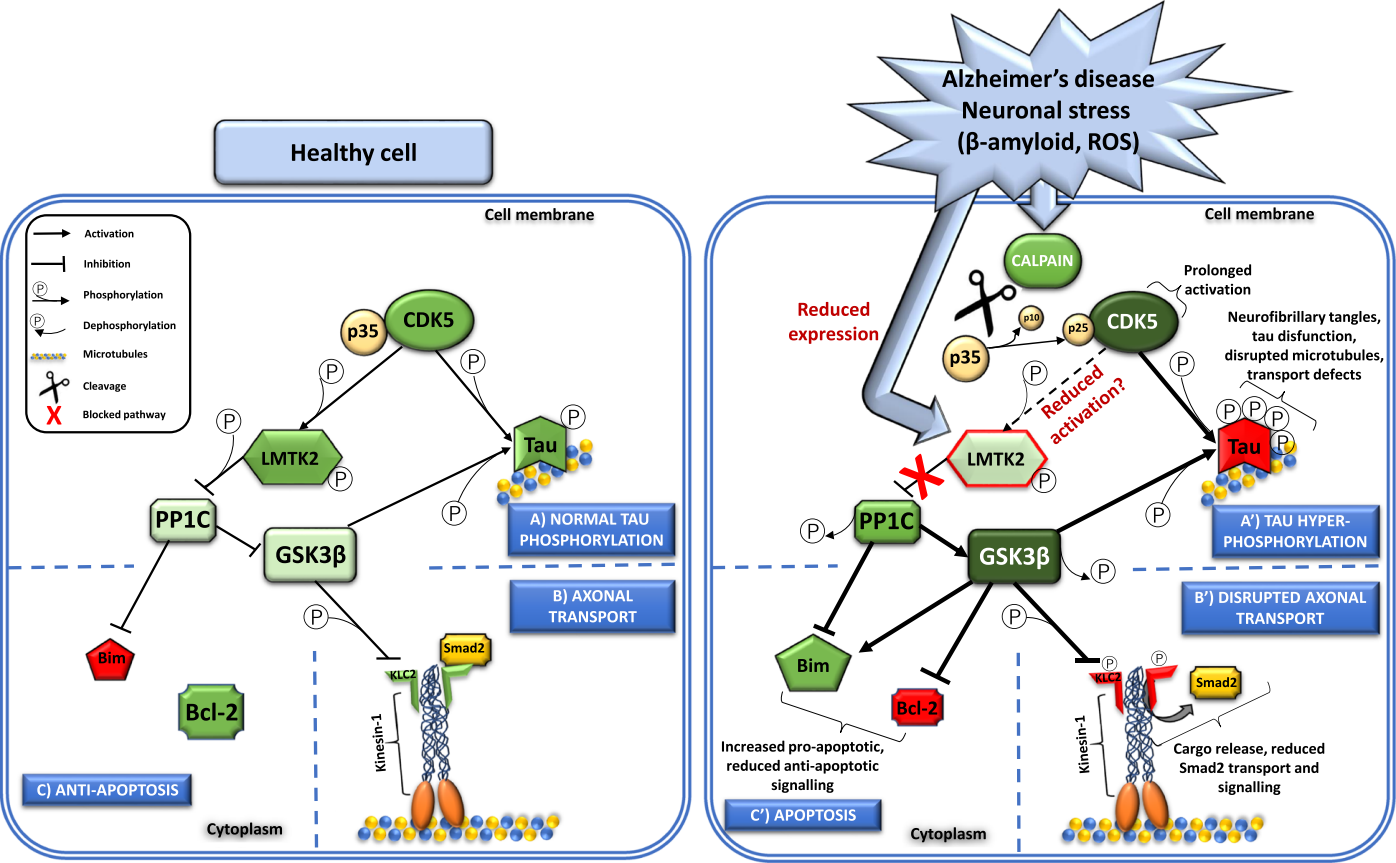
Potential LMTK2-related neurodegenerative mechanisms in Alzheimer’s disease. In *healthy cells*, CDK5/p35 phosphorylates and activates LMTK2. Activated LMTK2 inactivates PP1C by phosphorylating it which leads to increased inhibitory phosphorylation of GSK3β. In physiological conditions, (**A**) CDK5/p35 and GSK3β phosphorylates tau. (**B**) GSK3β also phosphorylates KLC2 to regulate kinesin-1 based transport while (**C**) PP1C inhibits pro-apoptotic factor Bim. *Alzheimer’s disease* related neuronal stress (e.g. β-amyloid, ROS etc.) induces calpain mediated cleavage of the CDK5 activator subunit p35 into p25 and p10. p25 prolongs CDK5 activity. Whether CDK5/p25 can activate LMTK2 in the same way as CDK5/p35 is not known. This and neuronal stress related reduced LMTK2 expression lead to blocked LMTK2 phosphorylation pathway. Disrupted LMTK2 phosphorylation pathway causes PP1C dephosphorylation and activation which in turn activates GSK3β by removing its inhibitory serine-9 phosphorylation. (**A’**) Overactivated GSK3β and CDK5/p25 abnormally hyperphosphorylate tau. Abnormal tau hyperphosphorylation disrupts tau function and microtubule network, affects axonal transport, and forms neurofibrillary tangles. (**B′**) Overactivated GSK3β phosphorylates KLC2 resulting in cargo release and disrupted Smad2 transport, and signalling. (**C′**) Overactivated GSK3β supresses anti-apoptotic Bcl-2 and increases the pro-apoptotic Bim levels, and signalling. Activated PP1 inhibits Bim however, it cannot counterbalance GSK3β mediated Bim activation and apoptosis. [Colour codes: Green symbols are functionally active, red symbols are inactive enzymes. Note that thickness of arrows and colour shades reflect activity levels where thin arrows/light colours represent low activity, and thick arrows/dark colours high activity. Abbreviations: Bcl-2 = B-cell lymphoma-2; Bim = Bcl-2-interacting mediator of cell death; CDK5 = cyclin-dependent kinase-5; GSK3β = glycogen synthase kinase-3β; KLC2 = kinesin-1 light chain 2; LMTK2 = lemur tyrosine kinase 2; PP1C = catalytic subunit of protein phosphatase-1; ROS = reactive oxygen species]

LMTK2 is involved in neuronal outgrowth and development, axonal transport, intracellular vesicle trafficking, and apoptosis [13, 22, 23, 48, 51]. Yet, the precise way how LMTK2 orchestrates these processes remains undiscovered. The determinant alterations in neurodegeneration are disrupted axonal transport, pathological accumulation/aggregation of disease-specific proteins and dysregulated apoptosis which together lead to neuronal loss [52, 53]. Taking into account of biological functions of LMTK2, there is a plausible link between LMTK2 and neurodegenerative processes (Fig. 1).

LMTK2 is enriched in the brain suggesting its crucial role in the central nervous system [16–19]. A recent genome-wide gene-expression study compared five amyloid and tau transgenic mice lines, and created a database [54]. Progressively decreasing *LMTK2* expression was found in the cortex and hippocampus predominantly in the tau mouse model (Tau P301L) during the disease development, and this reduction correlates with pathological changes. In contrast, in the cerebellum, increased *LMTK2* expression was detected and the animals did not show any cerebellar Alzheimer’s disease pathology. As it is detailed above, CDK5/p35 phosphorylates LMTK2 to activate it [15]. In neurodegeneration linked cellular stress conditions, such as β-amyloid overproduction or oxidative stress, calcium-dependent cysteine protease calpain is activated, and proteolytically cleaves p35 into p25 and p10 [55–58]. Owing to its longer half-life, p25 prolongs the active state of CDK5 resulting in increased phosphorylation of downstream targets [56, 59, 60]. However, if CDK5/p25 can phosphorylate LMTK2 in the same way as CDK5/p35 is not known. Although the number of experimental studies is certainly limited, based on the known biological functions and interacting partners, we attempt to provide a brief explanation how the previously reported reduced LMTK2 levels in Alzheimer’s disease animal model, and potentially disrupted phosphorylation cascade can contribute the disease pathology.

### Disrupted axonal transport

Physiological axonal transport is fundamental for maintaining the complex neuronal homeostasis. Neurons are polarised cells with majority of their proteins synthetized in the soma and require therefore well-organized intracellular transport to reach their targets. There are four vital actors in this play: microtubule tracks, molecular motor proteins, cargoes and energy in the form of ATP. Any disruption to this precise machinery perturbs axonal transport and causes aberrant accumulation of proteins, and organelles in different neurodegenerative pathologies in dementias, movement disorders and motor neuron diseases, for review see [52].

Kinesin-1 is a major molecular motor protein mediating axonal transport of several key cargoes such as mitochondria, amyloid precursor protein and synaptic vesicle precursors towards synapses [61–64]. Most functional kinesin-1 is a heterotetramer composed of two kinesin-1 heavy chains and two kinesin-1 light chains (KLCs). Kinesin-1 heavy chains move along microtubules while KLCs are mainly involved in cargo binding [62]. Phosphorylation of KLCs is an important cargo binding and releasing regulatory mechanism [61, 65, 66]. GSK3β can directly phosphorylate KLC2 to induce cargo release and supress kinesin-1 mediated transport [13, 65]. Involvement of CDK5 in GSK3β-dependent regulation of axonal transport was first described by Morfini et al. [66]. They have reported that CDK5 indirectly regulates GSK3β activity via PP1C. However, the nature of interaction (i.e. direct or indirect) between CDK5/p35 and PP1C was unclear [66]. Manser and co-workers have recently revealed that LMTK2 is the missing link between CDK5/p35, PP1C and GSK3β which brings these proteins together in one phosphorylation pathway to regulate kinesin-1 based axonal transport [13, 15]. In this novel signalling pathway, CDK5/p35 activates LMTK2 by phosphorylating it at serine-1418. Phosphorylated LMTK2 in turn reduces PP1C activity by phosphorylating it at threonine-320. Finally, phosphorylation of PP1C threonine-320 leads to increased inhibitory phosphorylation of GSK3β at serine-9 [13, 15]. Inhibited GSK3β is not able to phosphorylate KLC2 which promotes KLC2 binding to cargos, such as mothers against decapentaplegic homolog 2 (Smad2). Thus, LMTK2 is a negative regulator of KLC2 phosphorylation and LMTK2 activity promotes kinesin-1 based transport of Smad2 [13, 67]. Smad2 is a transcription factor which shuttles between the cytoplasm and nucleus, and is a crucial player in transforming growth factor-β (TGFβ) signalling pathway [68]. TGFβ induces Smad2 translocation into the nucleus where it regulates the expression of TGFβ-responsive genes [69]. siRNA knockdown of LMTK2 disrupts Smad2 binding to KLC2 and importantly, it also inhibits TGFβ-induced nuclear signalling of Smad2 probably due to affected Smad2 transport [13]. Reduced *LTMK2* gene-expression has been detected in an Alzheimer’s disease tau mouse model [54]. Additionally, altered TGFβ/Smad2 signalling has been observed in common neurodegenerative diseases, including Alzheimer’s disease [70, 71] suggesting that LMTK2 is not only involved in the regulation of kinesin-1 based transport but also can contribute the pathomechanism of neurodegenerative diseases by affecting axonal transport.

### Tau hyperphosphorylation

Neurofibrillary tangles are hallmark intraneuronal pathological features of Alzheimer’s disease. Neurofibrillary tangles consist of misfolded and abnormally hyperphosphorylated tau, a microtubule-associated protein [72, 73]. CDK5 and GSK3β are major tau kinases which are involved in tau hyperphosphorylation in vivo [74–77]. Although both CDK5 and GSK3β are sufficient to hyperphosphorylate tau, negative correlation has been revealed between their activities. It has been shown that increased CDK5 activity inhibits GSK3β by increasing its inhibitory phosphorylation at serine-9 [13, 15, 66, 78, 79]. Interestingly, CDK5 mediated GSK3β inhibition is age dependent. In young mice, GSK3β activity is reduced compared to aged mice where GSK3β activity is increased causing tau hyperphosphorylation [79, 80]. The exact mechanism of age dependently enhanced GSK3β activity and tau hyperphosphorylation is not known. One possible explanation is that the molecular breaking mechanism which inhibits GSK3β activity is defective. Progressively decreasing LMTK2 levels seen in an Alzheimer’s disease mouse model [54] could potentially lead to aberrant overactivation of GSK3β in aged mice. It is important to note that decreased LMTK2 levels and/or kinase activity leads to increased PP1C activity [13, 15]. It is also known that hyperphosphorylated tau filaments are able to activate PP1 [81–83]. PP1 can dephosphorylate tau on some residues which are abnormally hyperphosphorylated in Alzheimer’s disease [84, 85]. In fact, despite increased PP1 activity, tau remains hyperphosphorylated in Alzheimer’s disease suggesting that elevated tau phosphatase activity of PP1C cannot counterbalance increased GSK3β activity. The above hypothesis explains some aspects of tau hyperphosphorylation however, it needs further investigation.

### Apoptosis

A recent siRNA-based high-throughput screen has identified LMTK2 as a potential regulator of apoptosis [51]. siRNA mediated LMTK2 knockdown decreases anti-apoptotic B-cell lymphoma-2 (Bcl-2) and B-cell lymphoma-extra-large (Bcl-xL), and increases pro-apoptotic Bcl-2-interacting mediator of cell death (Bim) protein levels [51]. These LMTK2-associated alterations made cells more sensitive to toxic effects of apoptosis inducing ligands and other cytotoxic compounds. The effect of LMTK2 silencing on Bim levels is mediated by increased PP1C and GSK3β activity [51]. Bim levels are also decreased in the brain of Alzheimer’s disease patients and Bcl-2 is protective against Alzheimer’s disease-related insults [86, 87]. LMTK2 silencing was also accompanied by decreased Akt and extracellular signal-regulated kinase-1/2 (ERK1/2) activity [51, 88–91]. These changes can also contribute to apoptosis but the exact mechanism how LMTK2 can modulate Akt and ERK1/2 activity is not known. In essence, these results suggest that decreased LMTK2 levels can sensitise cells to cytotoxicity via affecting apoptotic and survival pathways, and it is consistent with the hypothesis that reduced LMTK2 levels may contribute to cell death in neurodegeneration.

According to our current knowledge, the majority of neurons are terminally differentiated cells [92]. However, certain noxious stimuli (e.g. oxidative stress, β-amyloid peptide, tau hyperphosphorylation) can induce aberrant cell cycle reactivation [20, 29, 93–95]. Since neurons lost their proliferative capacity, the abnormal cell cycle re-entry is interrupted by different regulatory mechanisms leading to apoptosis, for review see [95, 96]. Therefore, noxious stimuli which induce uncontrolled cell proliferation in other tissues could cause extensive cell death in neuronal tissue. In neurons, aberrant cell cycle reactivation upregulates cyclin D and cyclin E expression, and increases the activity of G_1_ and G_2_ phase CDKs [97–102]. Increased CDK activity results in retinoblastoma protein phosphorylation and concomitant E2F release in neurons [99, 102, 103]. E2F promotes the expression of pro-apoptotic genes and transactivates downstream cell cycle genes triggering the progression of the lethal apoptotic cycle [104]. Considering LMTK2 as tumour suppressor/pro-apoptotic protein, it is possible that E2F reduces LMTK2 levels by transcriptional silencing. Supporting this theory, several studies have reported decreased LMTK2 expression in both neurodegeneration and cancer [54, 105–109]. Nevertheless, the potential link between E2F and the *LMTK2* gene has yet to be explored.

## Conclusion and prospects

Prevalence of neurodegenerative diseases are exponentially increasing in our aging society. Considering that these frequent pathologies mostly affect the elderly, it is critical to provide an effective solution to this urgent issue. Although our knowledge is still insufficient, the latest studies point toward to the same direction: LMTK2 is involved in neurodegeneration (Table 1).

**Table 1:** Gene expression studies of *LMTK2* in neurodegeneration

Disease	Research model	Disease sample	Control sample	*LMTK2* gene expression in disease sample	Statistical significance	Reference
Alzheimer’s disease	Mouse tissue	Tau P301L cortex and hippocampus	Wild-type cortex and hippocampus	Decreased	Not known	[54]
Parkinson’s disease	Human tissue	Substantia nigra	Substantia nigra from controls	Decreased	Not known	[107]
Amyotrophic lateral sclerosis	Human embryonic stem cell-derived motor neuron	Neurons exposed to mutant SOD1 astrocyte conditioned medium	Non-treated neurons	Decreased	Not known	[108]
Huntington’s disease	Mouse tissue	DE5 (D9-N171-98Q) striatum	Wild-type striatum	Decreased	Yes (*p* = 0,025)	[109]

Despite the limited amount of studies in the field, LMTK2 seems to be mis- and downregulated in Alzheimer's, and other neurodegenerative diseases. Therefore, manipulation of the protein level could be a promising novel therapeutic target. A recent study has identified a 2-O-Tetradecanoylphorbol-13-acetate (TPA) responsive element in *LMTK2* gene [110]. TPA is a synthetic PKC activator with ability to increase LMTK2 expression. The mechanism requires activator protein-1 transcription factor complex binding to *LMTK2* promoter region in which the complex is trans-activated by protein kinase C [110]. These results are probably valuable but certainly not sufficient for drug development. Thus, further comprehensive investigations are essential to reveal and to understand the function of LMTK2 in neurodegenerative processes.

## Abbreviations

Bcl-2: B-cell lymphoma-2
Bcl-xL: B-cell lymphoma-extra-large
Bim: Bcl-2-interacting mediator of cell death
CDK5: Cyclin-dependent kinase 5
CFTR: Cystic fibrosis transmembrane conductance regulator
ERK1/2: Extracellular signal-regulated kinase-1/2
GSK3β: Glycogen synthase kinase-3β
KLC: Kinesin-1 light chain
LMTK2: Lemur tyrosine kinase 2
MAPK: Mitogen-activated protein kinase
NGF: Neuronal growth factor
PKC: Protein kinase C
PP1: Protein phosphatase-1
PP1C: catalytic subunit of PP1
PxxP: proline-(any residue-any residue)-proline
SH3: Src homology 3
Smad2: Mothers against decapentaplegic homolog 2
TGFβ: Transforming growth factor-β
TPA: 2-O-Tetradecanoylphorbol-13-acetate
VTF: Valine-threonine-phenylalanine
WWY: Tryptophan-tryptophan-tyrosine
